# How do pharmacists in English general practices identify their impact? An exploratory qualitative study of measurement problems

**DOI:** 10.1186/s12913-018-3842-y

**Published:** 2019-01-14

**Authors:** Georgios Dimitrios Karampatakis, Kath Ryan, Nilesh Patel, Wing Man Lau, Graham Stretch

**Affiliations:** 1 School of Pharmacy, University of Reading, Whiteknights Campus, PO Box 226, Reading, RG6 6AP UK; 2 Ealing GP Federation, 179C Bilton Road, Perivale, Greenford, Middlesex, UB6 7HQ UK; 3 School of Pharmacy, The Faculty of Medical Sciences, Newcastle University, Newcastle upon Tyne, NE1 7RU UK

**Keywords:** Pharmacists in general practice pilot, England, Impact, Activity codes, Qualitative study

## Abstract

**Background:**

In England, there is an ongoing national pilot to expand pharmacists’ presence in general practice. Evaluation of the pilot includes numerical and survey-based Key Performance Indicators (KPIs) and requires pharmacists to electronically record their activities, possibly by using activity codes. At the time of the study (2016), no national evaluation of pharmacists’ impact in this environment had been formally announced. The aim of this qualitative study was to identify problems that English pharmacists face when measuring and recording their impact in general practice.

**Methods:**

All pharmacists, general practitioners (GPs) and practice managers working across two West London pilot sites were invited, via e-mail, to participate in a focus group study. Appropriately trained facilitators conducted two audio-recorded, semi-structured focus groups, each lasting approximately 1 h, to explore experiences and perceptions associated with the KPIs. Audio-recordings were transcribed verbatim and the data analysed thematically.

**Results:**

In total, 13 pharmacists, one GP and one practice manager took part in the study. Four major themes were discerned: inappropriateness of the numerical national KPIs (“whether or not we actually have positive impact on KPIs is beyond our control”); depth and breadth of pharmacists’ activity (“we see a huge plethora of different patients and go through this holistic approach - everything is looked at”); awareness of practice-based pharmacists’ roles (“I think the really important [thing] is that everyone knows what pharmacists in general practice are doing”); and central evaluation versus local initiatives (“the KPIs will be measured by National Health Service England regardless of what we think” versus “what I think is more pertinent, are there some local things we’re going to measure?”).

**Conclusions:**

Measures that will effectively capture pharmacists’ impact in general practice should be developed, along with a set of codes reflecting the whole spectrum of pharmacists’ activities. Our study also points out the significance of a transparent, robust national evaluation, including exploring the needs/expectations of practice staff and patients regarding pharmacists’ presence in general practice.

## Background

The concept of having pharmacists employed in general practice is increasingly being investigated worldwide. Countries such as Australia, Canada, New Zealand, Malaysia and the USA have formally designed national programmes incorporating a non-dispensing pharmacist (involved in patient-facing activities beyond traditional medication dispensing) into general practice teams [1–5]. Some characteristic examples of services that general practice-based pharmacy teams carry out are: in depth face-to-face medication reviews (i.e. optimising treatment by stopping, amending or initiating medication guided by the patient’s current medical condition and the most recent guidelines - consideration is given to contra-indications, cautions and interactions) either inside the practice or in patients’ homes; updating medical records to reflect patients’ latest medications; education of practice staff around novel trends in pharmacotherapy; responding to general practitioners’ (GPs’) medication-related queries; quality assurance services to improve prescribing and medication use in the practice (e.g. relevant audits); prescribing tasks (including management of repeat prescriptions); and clinics for certain long-term conditions, such as hypertension, diabetes and asthma [6–8]. The provision of pharmacy services in general practice (e.g. medication reviews; initiation/adjustment of medications; adherence assessment; anticoagulation clinics; health/lifestyle advice; drug monitoring activities) has been found to significantly improve patient outcomes (e.g. systolic and diastolic blood pressure; medication adherence; glycosylated haemoglobin; low-density lipoprotein and total cholesterol levels) and patient safety [9]. It is also reported that pharmacy services in this setting can result in considerable cost savings to national healthcare systems via the prevention of hospital admissions and decreased drug expenditure through the optimisation of medication use [10]. Pharmacists’ work in general practice is additionally perceived to lead to considerable reductions in the workload of GPs [11, 12]. Furthermore, the presence of a pharmacist in general practice is often seen as an opportunity for following “precision medicine” patterns by offering patients individualised pharmacotherapy regimens and reducing unnecessary polypharmacy (i.e. concurrent use of multiple medications) [13]. Despite the described benefits, GPs’ reluctance to accept clinical interventions by pharmacists originating from historical inter-professional barriers (often characterised as “turf” protection) [14–17], along with patients’ unfamiliarity with a pharmacist’s role in this environment [18, 19], might significantly hinder pharmacists’ integration and subsequent utilisation within general practice. Therefore, it is essential that a pharmacist’s impact in this setting is measured and presented (through local evidence) to practice staff, policymakers and the general public [20, 21].

In England, there is a current shortage of approximately 8000 GPs and an oversupply of newly qualified pharmacists with excess numbers estimated to be between 11,000 and 19,000 within the next 20 years [22]. To address the present needs in the primary care workforce, National Health Service (NHS) England along with Health Education England (HEE), the Royal College of GPs (RCGP) and the British Medical Association’s GP Committee (GPC) are working in collaboration with the Royal Pharmaceutical Society (RPS) on a 4 year pilot to test the role and the effectiveness of clinical pharmacists in general practice [23]. This pilot is part of a larger national scheme focusing on building the future primary care workforce [24]. The main aim of the pilot is to reduce the workload of overburdened GPs, enabling them to focus on activities where they are most needed (e.g. diagnosis/management of complex patient cases), and offer patients greater access to health services and checks [25]. Within this context, NHS England will partially cover the expenses of co-locating a pharmacist as an equal member of the multidisciplinary team in the general practice environment. The pilot was announced in July 2015 with a budget of £15 million and involved 250 pharmacists [26]. In October 2015, NHS England increased the investment to £31 million which has involved more than 490 pharmacist posts across 90 sites which translates to approximately 698 practices in England [27, 28]. A pilot site is defined as a number of general practices, usually from the same geographical area, which participate in the national pilot scheme as part of the same organisation such as a GP Federation (i.e. a group of practices working together within their geographical area as part of a collective entity). In April 2016, a further £112 million was announced to support 1500 additional pharmacists in general practice by 2020 [29]. At present, the pilot serves over seven million patients and it is estimated that by 2020 a further six million patients will be covered by allocation of at least one clinical pharmacist per 30,000 population [30, 31].

The pilot is expected to be evaluated using Key Performance Indicators (KPIs) so that success and learning is identified and reported [32]. Currently, there are ten national KPIs based on numerical components (e.g. increase in total number of medication reviews) and two survey-based KPIs (requiring patient and GP surveys). Table 1 gives an overview of the national KPIs. For the numerical KPIs, the evaluation plan requires the practice pharmacists to record their day-to-day work on the clinical computer systems (SystmOne, EMIS and INPS Vision are the main computer systems in general practice in the UK). This could be done by using pre-defined electronic activity codes. Activity recording will enable a central investigation of the pilot outcomes by comparing baseline data (gathered at the initial stages or before pharmacists’ integration) with data collected well after the inclusion of pharmacists. Outcomes will then be audited against the KPIs.

**Table 1:** Overview of the national Key Performance Indicators (KPIs)

Numerical KPIs	
• Number of patient appointments with: General practitioner (GP), Practice Nurse, Clinical Pharmacist, Health Care Assistant/Advanced Nurse Practitioner • Impact on the percentage of patients who met the achievement indicator within the relevant Quality and Outcomes Framework - QOF (increase in the average QOF score) • Increase in total number of medication reviews • Decrease in the percentage of medication reviews undertaken by GPs • Increase in the total number of patients supported to develop care and support plans, including self-management • The rate of Accident & Emergency (A&E) attendances per 1000 patients on GP register • Rate of emergency hospital admissions for selected long-term conditions as a proportion of patients per GP practice • Reduction in the number of patients attending ≥15 appointments with a GP over the previous 2 years by age group (0–9, 10–19, 20–39, 40–59, 60–69, 70–89, 90+) • Reduction in antibiotic prescribing rate (versus national rate per STARPU - Specific Therapeutic Group Age-sex weightings Related Prescribing Units - a weighting system that takes into account the types of people receiving treatment within a specific therapeutic group in order to compare drug use between National Health Service organisations and practices) • Reduction in prescribing rate of anti-psychotic medications for patients with dementia or learning disabilities	
Survey-based KPIs	
• Patient satisfaction survey (patient experience) • GP survey (impact on workload, time, utilisation, job satisfaction)	

Although UK pharmacists have occasionally provided services in general practice in the past [33, 34], this is the first time that NHS England has tried in a formal way to implement and test the role of pharmacists in this setting. Despite the existence of central measures (i.e. national KPIs), as yet (2018) no national comprehensive evaluation of pharmacists’ impact in the general practice setting has been formally announced [35]. Therefore, our purpose with this qualitative study was to explore perceptions around the KPIs in two pilot sites in West London and identify problems (if any) pharmacists experience in measuring and recording their input. We anticipate that our findings and practical recommendations will be useful for national policymakers, professional bodies and pilot sites regarding what relevant actions should be taken to assist pharmacists in identifying and demonstrating their impact in the English general practice environment.

## Methods

### Study design

A qualitative design was chosen for the study to understand participants’ views in depth. Semi-structured focus groups, rather than individual interviews, were followed so that participants had the opportunity to collectively interact and to freely express their own ideas for discussion by the group.

### Setting

All participants were recruited from two West London GP Federations (both of which constituted pilot sites). These sites were chosen for the study as they both have working connections with the organisation of the research team. At the time of the study, one Federation had eight practices participating in the pilot (employing 36 GPs, nine managers and serving approximately 72,000 patients) and the other Federation had seven practices participating in the pilot serving 60,000 patients. Each Federation employed seven pharmacists who each undertook approximately 40 to 60 face-to-face patient appointments per week, thus, every pharmacist saw between 160 and 240 patients monthly. The pharmacy teams provided a variety of services including face-to-face medication reviews in the practices and in domiciliary settings including surrounding residential aged-care and nursing homes, for example, managing polypharmacy, optimising medications and performing patient monitoring activities; telephone consultations with patients, for example, managing minor ailments such as the common cold; clinics for long-term condition management (e.g. asthma/hypertension/anticoagulation/diabetes); answering GP and patient medication queries; reconciling discharge summaries; completion of prescribing audits; and organising practice education sessions (e.g. updating practice staff on new drugs). They also contributed to prescribing including signing repeat prescriptions. Prescriptions were processed either on site or electronically and authorised only by pharmacists who had completed an independent prescribing course.

### Participants and recruitment

To elicit representative and realistic views, only people directly involved in the pilot project (all pharmacists, GPs and practice managers in the two Federations) were invited to take part in the study. One very senior pilot pharmacist was excluded from participation because they are part of the research team and also to avoid discouraging less senior colleagues from expressing their honest views during the discussion. Invitation was via e-mail sent by the lead pharmacist of each of the pilot sites, on behalf of the research team. This e-mail attached an Invitation Letter, a Participant Information Sheet (describing the nature and the process of the study in detail) and a Consent Form. The e-mail also included the time and the venue for the focus groups and asked participants to contact either the lead pharmacist or a member of the research team (GDK) if they wanted to take part in the study. Potential participants had 1 week to decide whether or not to participate in the study. No follow-up, reminding e-mails were sent. Participation was voluntary and no monetary incentives were provided.

### Data collection

Data collection was divided into two parts over a 2 h period. During the first part, which lasted approximately 1 h, a Power-point presentation was given by the researchers to remind the participants of the KPIs at national level and to emphasise the importance of the measurement of pharmacists’ involvement in general practice. Additionally, the aim of the study was repeated to ensure that participants fully understood the study process. As the researchers were not known to participants, this preliminary seminar also acted as a rapport building session between researchers and participants (e.g. researchers had the chance to introduce themselves and explain their research interests). Participants had the opportunity to ask questions and put forward ideas for discussion before taking part in the focus groups. The second part of the session was the actual focus group discussions. The researchers split the 15 participants into two focus groups so that each of the participating pilot sites had equal representation in each discussion group. The composition of one focus group was eight participants (all pharmacists), while the composition of the other focus group was seven participants (five pharmacists, one GP and one practice manager). The two focus-groups were conducted concomitantly in different meeting rooms in one general practice which is part of one of the pilot sites. Written consent was obtained from all participants just before participation. The focus group facilitators talked as little as possible during the session, simply adding prompts to keep the discussion on topic. The participants were asked to comment on their thoughts and experiences of the national KPI list, a set of local KPIs developed by the pharmacy team of one of the recruited pilot sites and a list of activity codes extracted from one of the two largest computer systems being used in general practice. In addition, participants were asked to report any additional KPIs that might be useful within their own scope of practice and any day-to-day problems they were experiencing with the measurement of their input. The full focus group schedule (see Table 2) was pilot-tested on the lead pharmacist and pharmacy technician of one of the recruited pilot sites. All areas of the schedule were fully covered during the discussion and the facilitators ensured all participants had equal opportunity to express their opinion. Each of the focus groups lasted approximately 1 h (discussion was completed only when participants did not have anything else to add) and both were audio-recorded with consent from the participants. The facilitators were also keeping field notes when needed.

**Table 2:** Focus group schedule

Intro (setting the stage)	Questions	Closing
Each facilitator to introduce themselves Re-iterate purpose of the focus group Go over how the focus group will work, including reminder that session will be recorded Get participants to sign the consent form (with demographic details: registration year, years in general practice, role)	Do you have any questions before we start? Looking at the national Key Performance Indicators (KPIs), are you happy with them? Why and which ones are you not happy with? List of local KPIs: what do you think of these? Are there any additional KPIs that you think would be useful in your own scope of practice? With the KPIs we have agreed upon, what codes do we need to look at for each KPI? (go over each KPI, get an agreement on codes from the list provided and then move on to the next KPI) Probes: Can you give me an example of that? You mentioned …………… Would you tell me more about this? Can you explain that a bit more? Can you be a bit more specific about that?	Finalise any points about KPIs and codes Ask if any final questions Let people know how helpful they have been Make them aware of how they can reach researchers if they have any questions or additional thoughts

Two members of the research team (GDK and NP) acted as the facilitators of the focus groups and collected all data. Both facilitators have a pharmacy background (NP holds a Doctor of Philosophy - PhD - and serves as a lecturer in pharmacy practice whereas GDK is a doctoral research student). Both have experience in qualitative research and have undertaken previous training in focus group techniques.

### Data analysis

Audio-recordings of the focus group discussions were transcribed verbatim by the researchers (GDK transcribed the audio-recordings and the accuracy of transcription was verified by the rest of the research team) and thematically analysed. Thematic analysis was chosen as it is an intuitive interpretive process, allows for categories to be discerned directly from the data and enables the formation of trustworthy conclusions accounting for the whole range of individual participant experiences [36]. No theoretical framework was applied to the analytical process as the focus of this study was data driven to practically inform policymakers, pilot sites and pilot pharmacists, not to examine any behavioural changes or to interpret perceptions. Analysis was not done separately for each focus group (data from both groups was gathered and each pharmacist was coded, for example, Pharmacist 1, 2, 3 etc.). An inductive approach was followed [37]. The six phases of thematic analysis as described in the method of Braun and Clarke [38] were applied (familiarisation, coding, theme searching, theme reviewing, theme defining and naming, producing the report). The coding process was done manually (codes were annotated on the transcripts’ margins). At first, all of the data was systematically coded by GDK generating as many potential codes as needed (i.e. one single code for every different concept/idea identified). Coding was verified by the rest of the research team prior to generating categories and eventually themes. Then, all codes were transferred to a Word® document. Data identified by the same code was collated together and all different codes were sorted into potential categories (each category was highlighted with the same shading). After that, categories were re-examined and collapsed into potential themes with associated sub-themes. Then, the potential themes were re-assessed and re-organised. Finally, the whole research team together reviewed, refined and named the themes. Participants’ feedback on the transcripts or the summarised final findings was not sought.

## Results

Fifteen people (13 pharmacists including six independent prescribers, one GP and one practice manager) participated in the two focus groups. No participants withdrew during or after the focus groups. Table 3 provides an overview of the range of the participants’ demographics rather than the exact details so that anonymity is maintained. Clinical Commissioning Groups (CCGs) in the UK are clinically-led legal bodies responsible for the commissioning of healthcare services for their local area.

**Table 3:** Participants’ demographics

	Time in general practice	Time as qualified professionals	Background before joining general practice
Pharmacists (13)	2 months to 4.5 years	4.4 to 29 years	Hospital pharmacy (5) Clinical Commissioning Group (CCG) work (2) Community pharmacy (3) Unknown (3)
General Practitioner (1)	More than 30 years	More than 30 years	Not applicable
Practice manager (1)	More than 10 years	Not applicable	Not applicable

Free discussions were observed throughout the focus groups.

Four overarching themes were discerned during analysis of the focus group transcripts: inappropriateness of the numerical national KPIs; depth and breadth of pharmacists’ activity; awareness of practice-based pharmacists’ roles; and central evaluation versus local initiatives.

### Inappropriateness of the numerical national KPIs

The numerical national KPIs were believed to be unsuitable in identifying pharmacists’ input into general practice, for various reasons.

Participants claimed that KPIs are mostly designed according to economic priorities (e.g. savings in NHS resources) and that they do not specifically target pharmacists’ work. Consequently, it is not within the pharmacist’s remit to have an impact on most of the national KPIs and, also, any effect on a particular KPI can be attributed to different people’s input rather than to pharmacists specifically.

*Some of these KPIs, we have no control over. For example, patients attending more than 15 appointments, have we got any control over that? I don’t think so.* (Pharmacist 11)

*It would be very difficult to evaluate at which part of the path the clinical pharmacist’s role comes into play, because managing A&E [Accident & Emergency] admissions is disseminated from people involved into different clinical roles.* (Pharmacist 10)

Some of the KPIs (e.g. decrease in medication reviews undertaken by GPs) require coding by more than one healthcare professional (apart from pharmacists) and, subsequently, the existence of (baseline) data for these cases is highly dependent on the degree to which different people record their work.

*They [GPs] are not using the review codes, there is no way you can see what activity they’re doing, what activity we are doing, so in decreasing the percentage of medication reviews done by GPs, you’re not going to be able to get a sensible bit of baseline data.* (Pharmacist 2)

Frequent unintentional tick-box exercises by GPs, without a previous in-depth investigation of a patient’s medication problems, was thought to negatively affect baseline data by wrongfully elevating activity levels for GPs and thus reducing the visibility of pharmacists’ input.

*The doctors do medication reviews so mainly a tick box exercise, where they might say, “Oh, I can’t find medicines, fine, tick” and that’s their total baseline numbers, whereas we might do medication reviews and spend half an hour and go into a lot of detail. And that would be like one medication review.* (Pharmacist 6)

According to participants, the KPIs do not account for quality indicators around pharmacists’ work (e.g. depth, effectiveness or influence of intervention/consultation) as most of them are purely based on numerical aspects (such as appointment numbers, increase in the total number of medication reviews etc.) and not on any value components.

*What I’m doing in terms of a consultation is clicking on something. So, for example, I do a care-plan, it’s just the number isn’t it? I’m just generating numbers, it doesn’t show you quality.* (Pharmacist 8)

The national KPI which relates to the Quality and Outcomes Framework (QOF - a voluntary programme for English general practices with the purpose of motivating and rewarding clinical excellence) indicators was deemed by the majority of participants to be irrelevant because QOF measures were thought not always to be based on the latest updated guidelines of the respective health authorities. For example, QOF measures related to diabetes were believed to often include a glycosylated haemoglobin target level significantly different from the one reported on the national diabetes guidelines.

There were also concerns that data collected for the KPIs might, in parallel, be used for comparing the performance of individual pharmacists across the national pilot.

Overall, participants claimed that the whole KPI concept treats pharmacists in general practice unfairly. Pharmacists’ continued presence in this setting, they thought, is dependent on whether or not their activities have positive outcomes on certain measures. They thought the role of GPs, in contrast, is well established and secured over and above any impact on any indicators.

*Whether or not we actually have positive impact on KPIs is beyond our control, but it is providing a staffing base to do roles which the practice needs. I think it’s not particularly fair the fact that you’re not looking at giving them [general practices] an extra GP and then, for example, seeing admission rates fall, saying “Oh, after all, a GP does have a job role”.* (Pharmacist 2)

Despite these general problems, participants recognised aspects of the KPIs where pharmacists could make a difference. For example, the development of comprehensive care-plans was perceived to be directly related to pharmacists’ expertise and, thus, a good means of showing impact on patients (e.g. clear administration schedules, instructions on when to seek pharmacist’s help, raised levels of patients’ understanding around their condition). Reducing unnecessary requests for antibiotic rescue packs, updating practice staff on the latest anti-psychotic medication guidelines and pre-empting frequent appointments and phone calls from high users of GP services were also suggested ways of impacting upon the respective KPIs. Finally, since many pilot sites have nursing homes attached to them, participants said there is an ongoing need for a national KPI accounting for practice-based pharmacists’ activities in nursing homes.

*We thought there may be specific applications of clinical pharmacy into nursing homes. And I still believe that these have been the case. Pilot sites cover 95% of the nursing home patients. I just wonder whether there’s something, or some things, we could be measuring around nursing homes, which would be meaningful.* (Practice manager)

### Depth and breadth of pharmacists’ activity

Practice-based pharmacists were confident that their activities, especially the medication-related ones, bring additional quality to the services provided in general practice and improve the standard of patient care. Pharmacists reported investing time when reviewing a patient, for example during a scheduled consultation for a medication review or a care-plan development, and following a holistic approach which is characterised by an in-depth investigation of every health problem a patient experiences regardless of whether it originates purely from medications or not (e.g. dealing with mental health, dexterity, mobility, lifestyle problems or other situations that individual patients might face).

*You can assume if a pharmacist is doing a medication review, then there is an inbuilt quality that is not otherwise there.* (Pharmacist 2)

*We see a huge plethora of different patients and go through this holistic approach - everything is looked at, including medications. In fact [sometimes] when we review a patient, medications play only a small part [in the review process] and the focus is on mobility, mental health, memory [problems], activities of daily living [*etc.*].* (Pharmacist 5)

Participants said, however, that pharmacists’ activities are not always being captured and recorded on the electronic systems of general practices, as the available activity codes do not often match actual tasks or they are not specific enough. Therefore, the current electronic codes fail to differentiate work and show all the different activities that pharmacists cover on a day-to-day basis.

*Our two pharmacists are just invaluable for the stuff they carry in their heads about medication interactions or complex things that you can phone and ask or e-mail. There’s no code for that. But it’s actually very, very important.* (GP)

*There is a general code about [medication] monitoring but not specifics about whether you have adjusted the medication because of the bloods, or checking bloods for monitoring. None of that is captured and I do that nearly every day.* (Pharmacist 1)

For this reason, participants referred to the need to investigate the range of pharmacists’ work across English general practices and produce a global list of activities widely expected to be carried out by pharmacists in this setting.

*We need a global list of activities - core activities of the clinical pharmacist - that [we] are expected to carry out and some other little bits and pieces that we do on a daily basis and around those shape the codes.* (Pharmacist 12)

Until then, a general pharmacist code (such as “Pharmacist” or even simply “P”) was thought to potentially act as a surrogate for activities that are currently not coded. In addition, as every pharmacist’s consultation or other action is automatically time- and name-stamped on the clinical computer systems, searching the system by name (name searches) was perceived to be a complementary method (to the conventional coding-dependent process) of getting an insight into the extent of work a pharmacist does.

### Awareness of practice-based pharmacists’ roles

Participants commented on the necessity of increasing the awareness of both primary care team members and patients around the role and the capabilities of a pharmacist co-located in general practice.

*I think the really important [thing] is that everyone [within the general practice] knows what pharmacists in general practice are doing. And it takes a bit of time. And there is a presumption that everybody knows what pharmacists have been doing. They don’t.* (GP)

As practice-based pharmacists are in a perfect position to link different professionals and act as the first point of reference in general practice, networking with other practice staff can provide pharmacists ample instances to communicate and promote their role.

*Your dieticians, your physios, the in-house smoking cessation services, all these people now come through us. So, we’re now dealing with all sorts of prescribing needs, including for care homes. So, it’s all these different angles, like the mental health reviews, they all come in [to the general practice] and you do the prescribing. So, you’re linking in with all of the teams and you’re their contact.* (Pharmacist 7)

Especially essential is the building of rapport with the local community pharmacists as this unifies patient care by reducing instances of conflicting interventions from pharmacists in different settings.

*I think a really legitimate KPI will be “contact with the local community pharmacy” and it is sort of starting and maintaining a relationship with the community pharmacies, so that’s about: how many are there and how many have we spoken to. And the ultimate goal must be to speak to all of them, to interact with, and make sure you [practice-based pharmacists] have a common language to talk [with the local community pharmacists].* (Practice manager)

A close working relationship with community pharmacists was also believed to offer practice-based pharmacists the opportunity to enhance the scope of Medicine Use Reviews (MURs - a service offered by UK community pharmacies which involves adherence-focused reviews with patients, mainly those with a targeted condition such a as asthma, diabetes etc., to confirm that they are taking or using their medications in an optimal way to derive maximum benefit from their therapy) by encouraging their colleagues in community pharmacy to report to the local general practice any outstanding clinical problems they identify. Furthermore, interacting with community pharmacists allows pharmacists in general practice to contribute to reducing medicines waste and thus positively impact overall costs to the NHS.

*It would be interesting to investigate the impact of having a pharmacist in a surgery on reducing waste of the NHS in terms of writing prescriptions, medication being out of date so then affecting the cost in the long-term. So, people keep bringing a bag to [community] pharmacy for disposal and I guess having a pharmacist now in the surgery may influence that by asking the community pharmacist to feedback the amount and the type of wasted medications for each patient where this is clinically relevant.* (Pharmacist 12)

Participants said that, as a means of showing self-development, it would be worthwhile for practice-based pharmacists to systematically survey levels of understanding, amongst patients and practice staff, around pharmacists’ roles in general practice.

*The [pilot] pathway does require us at some stage to do patient satisfaction survey and the GP survey. It would be useful for us and useful in terms of insurance and everything else, to say: “We’re actually developing ourselves”.* (Pharmacist 2)

Participants highlighted the need for a standard patient survey specific to pharmacists’ services. Adapting the form that GP trainees use for exploring patients’ views on their work and/or using the form of the “friends and family” test (i.e. a single item survey asking patients whether they would recommend the healthcare service they have received to friends and family members) were proposed as contemporary surrogates. The “friends and family” test, however, was thought to hardly distinguish those patients who accessed pharmacy services as it seeks feedback on the practice-based services in general, rather than on individual healthcare professionals.

*The problem with the “friends and family test” is that it is very hard to differentiate between a patient who’s gonna see a clinical pharmacist and a doctor. So, in order to use “friends and family” specifically for the clinical pharmacist you’d almost have to turn it off for everybody else.* (Practice manager)

Despite their importance, patient surveys are often associated with low response rates. Consequently, practice-based pharmacists often face difficulty in showing that they are proactive with the respective national KPI based on patient surveys.

*When giving patients the questionnaire, you could always quote [on the electronic system] that you’ve given this, so you can measure how many patients have received it. But you won’t know how many [patient surveys] you’ve got back.* (Pharmacist 9)

Participating pharmacists were also very concerned that patient surveys could contain a powerful bias as they might be dominated by negative views towards the practice-based pharmacist. Several problems were perceived to form reasons for potential patient dissatisfaction. There are instances where patients with a condition unsuitable for pharmacists’ knowledge are wrongfully being directed to the practice-based pharmacist (triage problems). As a result, the pharmacist is unable to perform a successful intervention and satisfy their needs or expectations.

*Patient satisfaction is quite tricky because it’s not always a fair opinion. Just from what I’ve seen, patients half the time they don’t know they’re coming to see you [practice-based pharmacist], so they’re instantly annoyed because they come with something that you can’t actually deal with because it’s been wrongly written in reception or something like that, so their satisfaction is going to be poor.* (Pharmacist 3)

Patients might negatively link contact with the practice-based pharmacist with undesired amendments to their therapy.

*When patients come in [to see the pharmacist in general practice] sometimes, they know they’re gonna switch something [medication] or stop it and they’re on the defense straight away and they’re not going to be satisfied [with practice-based pharmacists’ services].* (Pharmacist 7)

Patients who complain are usually keen to fill in surveys and this coupled with the fact that “thank-you” messages (expressed through cards, notes or presents) are not being formally recorded were perceived to further overshadow any positive views.

*The typically demanding people are those who will take the time then [after a consultation] to go on and complain [through patient surveys] about what’s happened.* (Pharmacist 2)

To overcome the bias of unfair negative attitudes and elicit more representative patient feedback, it was suggested that survey forms could be individually handed to those patients who consciously (i.e. aware of the practice-based pharmacist’s presence and the respective services provided) experience regular contact with the practice-based pharmacist.

*You could focus on getting some kind of a survey for those patients who you are seeing on a regular basis or they are coming in [to the general practice] just for a particular clinic [with the practice-based pharmacist] and so they know that they’re going to be seeing you [practice-based pharmacist] in the first place.* (Pharmacist 3)

Another identified potential bias is that the structure of the survey form does not take into consideration individual needs that sectors of the patient population have. For example, surveys are often available exclusively in an online version requiring computer skills thus large numbers of patients (e.g. elderly) might be prevented from participating.

*In each GP practice that’s a big problem historically with how feedback is given, I mean patient satisfaction surveys in GP surgeries. The NHS choices [around patient surveys] feed into a computer literate group of people, not typically the elderly with chronic diseases and it massively biases the sort of feedback.* (Pharmacist 2)

### Central evaluation versus local initiatives

Participants were certain that NHS England will nationally evaluate the whole pilot project to reveal outcomes for both general practices and patients.

*The KPIs will be measured by NHS England, regardless of what we think. I see it’s a perfectly legitimate thing for them to pull out of their national statistics these particular measures.* (Practice manager)

It was felt obligatory, therefore, for the general practices and the pilot pharmacists to adhere to the national KPIs (despite the inappropriateness of most of them for pharmacists), as NHS England will still follow the relevant measures to satisfy each national KPI.

*We do have to follow them [national KPIs] because they’re national. Some of these don’t apply to us but, yes, I think they [NHS England] are gonna be pulling out the [appropriate] figures [to satisfy each KPI], at the end.* (Pharmacist 11)

Practices, however, can additionally create local indicators (taking into consideration local priorities and needs) that will act as supplementary measures to the national KPIs.

*What I think is more pertinent, are the things - are there some local things? We’ve got a great opportunity between - among the two boroughs to say “Ok, let’s have a simple list of [local] KPIs which we agree we’re going to measure”, fantastic!* (Practice manager)

Within this framework of local actions, each practice could quantify the work carried out by pharmacists. Examples given were the numbers of medication reviews, therapy amendments or just numbers of patients who contacted the practice-based pharmacist.

*What would be more specific is to evaluate the work that we actually do, in addition to this [the national KPIs]. What would be more specific to each one of us as pharmacists is that our specific practice, maybe at the end of the year or month, measures our work, to say that “We’ve actually done this amount of work that has impacted the surgery by this much”.* (Pharmacist 13)

Participants reported that recording completed pharmacists’ tasks could show pharmacists’ involvement in duties that would otherwise have been performed by GPs or other practice staff. Showing a reduction in the workload of GPs was perceived to be the best way of showing pharmacists’ impact in general practice.

Pharmacists were very confident that their presence in general practice has positive outcomes for patients and practice staff and that they will maintain their employment in this setting even after the national pilot is over.

*One thing that’s interesting, they [NHS England] think they’ve come up with a novel idea, the pilot, whereas your pharmacists in practice get it. So, for decades people have known it’s worthwhile. Join the club! I was employed for the pilot and will be employed after the pilot, I’m sure, as will probably all of us because it’s already there - the role exists.* (Pharmacist 2)

The GP who participated in the focus group was also very optimistic about the pilot and reported that time-savings in GPs’ workload are already obvious.

*A practice-based pharmacist probably saves each GP at the practice an hour per day. I’m very enthusiastic about the idea [of pharmacists in general practice] and I see a real potential.* (GP)

## Discussion

Participants believed that the majority of the national KPIs are inappropriate and that pharmacists’ day-to-day efforts are not always being captured through the current electronic coding systems. The necessity of raising the levels of knowledge, amongst primary care staff and patients, about practice-based pharmacists’ services was highlighted. There was an expectation by participants for a central evaluation of the “pharmacists in general practice” pilot project. The value of creating local indicators was also noted.

Participants reported the inability of pilot pharmacists to show an impact on most national KPIs. In contrast, pharmacists in Australian general practices were able to show their impact within 6 months of their interventions (face-to-face patient consultations) [39]. The impact measure in the Australian study was the number of patient medication-related problems (MRPs), for example, inappropriate dose or drug interactions that pharmacists were able to resolve. Similarly, practice-based pharmacists of the IMPACT (Integrating Family Medicine and Pharmacy to Advance Primary Care Therapeutics) project in Ontario, Canada, showed within 1 year of their integration impact on the practice through their contributions around diagnosis (e.g. of untreated indications), prescribing (e.g. of the right drug for the patient’s condition) and education (e.g. increasing the awareness of patients around their medications/disease condition) [40].

Although KPIs have successfully been introduced in other countries in the past to investigate different dimensions of the impact of new pharmacy services (e.g. ward pharmacy services in New Zealand public hospitals [41]), England is a pioneer in implementing KPIs for pharmacists in the general practice setting. The English pilot was at the time of the study in its second year and our work shows that the available KPIs are inadequate for explicitly identifying and showing pharmacists’ impact in general practice. These national KPIs are not fit for purpose in that they do not properly reflect the definition that Fernandes et al. ascribed to the ideal KPI for clinical pharmacy services: “a measure that reflects quality, relates to pharmacist role and is supported by adequate evidence” [42]. Measuring the quality of a healthcare service, however, appears not to be a straightforward process and the implementation of quantitative measures often leads to a ‘quality versus quantity battle’ [43]. According to Avedis Donabedian, the quantity of clinical activities itself does not necessarily signify quality unless interventions, in a specific setting, are strongly associated with desirable patient outcomes (in which case the presence or absence of an activity can itself indicate good or bad quality, respectively) [44, 45]. Real impact of a healthcare service, therefore, links with quality which (in turn) relates to outcomes.

Our study emphasises the importance of professional relationships between practice-based pharmacists and other primary care staff. Jorgenson et al. in their guidelines for pharmacists in primary care settings refer to professional interactions as “one of the biggest factors that will dictate the success of the pharmacist” [46]. This is consistent with the perceptions of the Canadian pharmacists in the IMPACT project who refer to interacting and receiving support from other professionals in primary care as one of the most significant facilitators for a smooth integration into general practice [47]. The participants in our study especially noted the necessity of a strong link between practice-based and community pharmacists as a means of unifying patient care which has been echoed elsewhere [48].

Participants in the current study recognised the usability of satisfaction surveys, especially of those targeting patients or GPs. Τhe perceived causes of potential unfair patient discontent (expressed via surveys), however, identify some problems pharmacists in English general practices experience, some of which are fairly similar to those overseas practice-based pharmacists had to overcome. The triage and maintenance of a core of patients (i.e. a consistent number of patients who are aware of the practice-based pharmacists’ presence and who visit the practice on a regular basis just for a consultation with the co-located pharmacist) that could benefit from pharmacists’ skills seems to be a historical problem at the initial stages of pharmacists’ integration into primary care settings [49]. Moreover, associating pharmacists with therapy changes (patients sometimes report that the main reasons behind therapy changes are monetary, for example, introduction of a cheaper alternative medicine) is an established literature finding [18, 50]. For the English reality, the latter point could be explained as a feature of culture which is often GP-orientated and not always fully informed about what a pharmacist can or cannot do for the patient [51].

Pharmacists employed in various South-West English environments viewed the relief of GPs’ work pressure as a prerequisite for undertaking any general practice roles [52]. Similarly, GPs in another English qualitative study point out that pharmacists should “demonstrably reduce GPs’ workload” to disprove any negative perceptions amongst practice staff peers and successfully incorporate into general practice [53]. This opinion is congruent with the perceptions of our participants who deemed the shift in GPs’ workload as the greatest factor in showing pharmacists’ impact and success of the pilot.

The current study did not reveal any initial “outsider feeling” which was the case for Canadian pharmacists in this environment [54]. In contrast, our participating pharmacists were confident about the high standard of their activity and their continuing presence in general practice (post the national pilot). It should be mentioned, however, that though the vast majority of our participating pharmacists were relatively new in their current general practice roles within the pilot, some of them had previous experience (up to 4.5 years) of some sort of work in general practice. Therefore, this may have been the reason for different confidence levels between our pharmacists and the pharmacists in the Canadian study by Pottie et al. [54].

### Study strengths and limitations

To our knowledge, this is the first study investigating problems around the measurement of pharmacists’ input in English general practices. The results can be extrapolated to various pilot sites nationwide and might also be useful for overseas policymakers and practice-based pharmacists. The free and extensive discussions observed meant that participants’ views were understood in depth. The study also accounted for perceptions of other practice staff members apart from pharmacists as it had knowledgeable representatives from the GP and managerial groups who explored the topic from different angles. Our study achieved a very high participation rate for pharmacists since all eligible pharmacists from the recruited pilot sites participated in the focus groups and, thus, we obtained the full range of possible pharmacists’ views from these sites. Another strength is that demographic characteristics of participating pharmacists were quite broad and so the findings reflect different levels of experience, backgrounds and roles.

One of the study limitations is that the overall number of participants was small and originating only from two pilot sites. Therefore, there may be more or different problems with the measurement of pharmacists’ input arising from different models of interaction and practice that exist across the country (e.g. different ways of employing practice-based pharmacists or different make-up of the general practice team or different pharmacists’ roles or contributions/services or different patient populations or other local features). The research team, however, mixed the participants from the two sites during the focus groups to encourage trans-site interactions and a wide exchange of different experiences. The views from the GP and practice manager must be regarded as an indication only (i.e. GPs’ and managers’ views were not exhaustively explored) because there was only one participant from each group. The purpose of the study, however, was not to compare perceptions between different professionals but to understand the overall opinions in depth (quotes from the GP and practice manager were chosen as they reflected the opinions of the group). A reflexive method of data interpretation was followed throughout data analysis as the researchers ignored any personal experiences and results were collectively analysed and discussed. Some unavoidable instances of personal assumptions during categorisation of the data, however, might still exist. Finally, there might have been facilitation differences amongst the two focus groups which possibly translates to some divergence in the depth and breadth of topics explored in the discussions. Both facilitators, however, followed the same focus group schedule to ensure that all main questions were adequately covered.

### Implications for practice and research

Our findings contain several useful points for NHS policymakers (both at a national and local level) and practice-based pharmacists. The most pertinent points are summarised below.

#### Policymakers should

- Determine national measures (explicitly based on key pilot stakeholders’ opinions) that will ultimately mirror the quality of practice-based pharmacists’ services.
- Develop complementary local indicators as per the needs or goals of individual practices.
- Produce electronic activity codes encompassing the whole range of pharmacists’ activity (across different work models) to encourage a more consistent and effective coding of work.
- Consider including amongst the KPIs activities relating to pharmacists’ capabilities in nursing homes, MURs, medicinal waste and interactions with community pharmacies.
- Develop clearly defined policies and task descriptions for the whole multidisciplinary team (e.g. conditions under which a certain task is considered satisfactorily complete) to limit instances of tick-box exercises and gradually build sensible data for any comparisons to be made.
- Design and validate, in conjunction with patient groups, standardised patient surveys specific to practice-based pharmacists’ work.
- Arrange booking systems of the practices to filter patient cases that could benefit from contact with a pharmacist.

#### Practice-based pharmacists should

- Use effective means to strengthen their utilisation and professional networks, such as participating in all multidisciplinary team meetings, liaising with other practice staff and interacting with nearby community pharmacies.
- Increase their visibility to patients by engaging with Patient Participation Groups; adding their name/face to the Practice notice boards and websites; producing leaflets or waiting room videos.
- Survey patients with regular contact with a general practice pharmacist and ensure that all kinds of feedback are formally recorded.

Future research should include the perceptions of larger cohorts of pilot pharmacists practising in multiple locations across England to identify any further problems that different models of practice-based pharmacists might experience.

## Conclusions

The participants in this study thought that the national KPIs are not fit for the purpose of identifying and demonstrating pharmacists’ impact in general practice. Therefore, it is important that generalisable measures, specific to pharmacists’ roles and reflecting the effectiveness and depth of their work and expertise, are developed along with a set of new activity codes accounting for the whole spectrum of pharmacists’ responsibilities. Our findings also constitute a forceful call for a transparent, robust and fully supported, by all stakeholders, national evaluation of all aspects of the pilot, including exploring the needs and the expectations of patients and GPs regarding the presence of pharmacists in the general practice setting. Every feasible method should be employed to extract the required data for the evaluation, including quantifying pharmacists’ work at a local level and obtaining data through searching the electronic systems by pharmacists’ names. An acknowledged evaluation will unveil strengths and limitations of the national pilot and will explicitly determine any further expansion of pharmacists’ roles and integration into this environment.

## Abbreviations

A&E: Accident & Emergency
CCG: Clinical Commissioning Group
GP: General Practitioner
GPC: British Medical Association’s General Practitioners Committee
HEE: Health Education England
IMPACT: Integrating Family Medicine and Pharmacy to Advance Primary Care Therapeutics
KPI: Key Performance Indicator
MRP: Medication-Related Problem
MUR: Medicine Use Review
NHS: National Health Service
QOF: Quality and Outcomes Framework
RCGP: Royal College of General Practitioners
RPS: Royal Pharmaceutical Society
STARPU: Specific Therapeutic Group Age-sex weightings Related Prescribing Units
